# Antimicrobial Effect of *Ocimum gratissimum* L. Essential Oil on *Shewanella putrefaciens*: Insights Based on the Cell Membrane and External Structure

**DOI:** 10.3390/ijms241311066

**Published:** 2023-07-04

**Authors:** Yao Xie, Chi Zhang, Jun Mei, Jing Xie

**Affiliations:** 1 College of Food Science & Technology, Shanghai Ocean University, Shanghai 201306, China; m210311052@st.shou.edu.cn (Y.X.); m210300872@st.shou.edu.cn (C.Z.); 2 Key Laboratory of Aquatic Products High Quality Utilization, Storage and Transportation (Co-Construction by Ministry and Province), Ministry of Agriculture and Rural Affairs, Shanghai 201306, China; 3 National Experimental Teaching Demonstration Center for Food Science and Engineering, Shanghai Ocean University, Shanghai 201306, China; 4 Shanghai Engineering Research Center of Aquatic Product Processing and Preservation, Shanghai 201306, China

**Keywords:** *Ocimum gratissimum* L. essential oil, *Shewanella putrefaciens*, antibacterial effect, biofilm, cell membrane, active compounds

## Abstract

The main objective of this study was to assess the in vitro antibacterial effectiveness of *Ocimum gratissimum* L. essential oil (OGEO) against Shewanella putrefaciens. The minimum inhibitory concentration and minimum bactericidal concentration of OGEO acting on S. putrefaciens were both 0.1% and OGEO could inhibit the growth of S. putrefaciens in a dose-dependent manner. The restraint of the biofilm growth of S. putrefaciens was found in the crystal violet attachment assay and confocal laser scanning microscopy. The disruption of cell membranes and exudation of contents in S. putrefaciens with OGEO treatment were observed by scanning electron microscopy, hemolysis and ATPase activity. The results demonstrated that OGEO had a positive inhibitory effect on the growth of S. putrefaciens, which primarily developed its antibacterial function against S. putrefaciens by disrupting the formation of biofilms and cell membranes. This study could provide a new method of inhibiting the spoilage of food in which the dominant spoilage bacteria are S. putrefaciens.

## 1. Introduction

Shewanella putrefaciens is recognized as the chief spoilage bacteria in fish [1]. It remains a significant threat to fish even in refrigerated conditions [2]. S. putrefaciens can produce high levels of trimethylamine, which can cause the fish to develop an undesirable flavor. In addition, mass degradation likely leads to the forming of the other amine compounds [3]. Biofilms produced by bacteria can act as reservoirs for spoilage bacteria in food and lead to the economic losses from food spoilage [4]. Biofilms are complicated communities of cells made up of bacteria. One of the reasons for the resistance of bacteria to disinfectants and antimicrobial agents is that extracellular polymers encase the bacteria, enhancing their resistance to external stimuli. During the synthesis of the biofilms, bacteria are encapsulated in an extracellular matrix consisting of proteins, polysaccharides and nucleic acids to form a three-dimensional structure [5]. Therefore, the bacteria become significantly more resistant against antimicrobial agents.

Antibiotics are rapidly increasing in resistance and other options need to be explored, such as natural products that may inhibit microorganisms ‘in vitro’ and in the ‘in situ’ gas phase, have antibiofilm effects and may act as biological antimicrobial agents [6]. The chemical antimicrobial agents are more harmful to the human body and natural preservatives have become a new trend for antibacterial agents. Essential oils could inhibit bacterial growth to control foodborne pathogens and fish spoilage [7]. Many research studies have demonstrated that EO are an effective antibacterial agent. In general, the effect of this EO is closely linked to the ability to permeabilize and/or disrupt membrane integrity, resulting in the leakage of intracellular material [8]. *Ocimum gratissimum* L. essential oil (OGEO) is rich in phenolic compounds which have potential antibacterial, antioxidant and insecticidal activity [9]. Eugenol and caryophyllene are the main active ingredients of OGEO. The antibacterial effect of eugenol has been verified. Eugenol can disrupt the cell membrane structure and change the permeability of bacteria [10]. The antibacterial activity of caryophyllene has been noted in a previous study [11]. Bassolé et al. [12] confirmed that OGEO inhibited various bacteria, such as Staphylococcus aureus, Enterococcus faecalis, Escherichia coil, etc. OGEO is classified as a GRAS (generally recognized as safe) compound and is allowed to be used as a food additive in China, USA and EU [13]. It was reported that rats given 1 g/kg body weight (1%) all survived with no abnormalities in their general behavior [14]. In this experiment, the maximum concentration of OGEO was also 0.1%, thus causing no harm to the consumer’s health. The antibacterial and antibiofilm activity of OGEO against S. putrefaciens should be investigated to assess the use of OGEO as a natural preservative. Consequently, the current study aimed to evaluate the biofilm inhibition mechanism of OGEO against S. putrefaciens. The inhibition effects of OGEO-treated S. putrefaciens were determined by the biofilm formation, field emission Fourier transform infrared spectroscopy (FTIR) analysis, SEM, extracellular polymers, hemolysis experiments, etc.

## 2. Results and Discussion

### 2.1. Major Compounds Detected in OGEO

A total of 42 compounds were detected completely by chromatography–mass spectrometry (GC-MS) (Table 1). OGEO was mainly composed of eugenol (51.88%) and caryophyllene (9.57%), which belong to antibacterial ingredients. Eugenol and caryophyllene were also shown to be the main components of basil essential oil in a study by Torpol et al. [15]. Shubham Srivastava et al. [16] reported that eugenol content in OGEO could reach 37.8–45.7%. Among other basil essential oils where eugenol is the first major component, the second major components are terpinolene [17], α-ocimene [18], germacrene-D [19] and citronellal [20]. Previous studies had also reported that the major component of basil essential oil is methyl vanillin (86.60%) [21]. Numerous studies on the composition of OGEO have shown the great diversity of its composition. The chemical types vary from many regions of the world. Therefore, the differences in OGEO composition may be connected with the type of basil, the part of basil, the growing environment and the extraction method.

### 2.2. Minimum Bactericidal Concentration (MBC) and Minimum Inhibitory Concentration (MIC) Value of OGEO

The MBC and MIC values of OGEO against *S. putrefaciens* were both 0.1% (1 μL/mL). The value of MIC of OGEO was identical to that of MBC. Such a result also occurred in the study of Krstev et al. [22]. In their study, OGEO inhibited the growth of several strains but the strongest inhibitory effect was observed in *Staphylococcus aureus* with an MIC of 0.75 µg/mL (0.075%) and an MBC of 1.5 µg/mL (0.15%). The MIC value for OGEO against *Salmonella typhimurium* (Gram negative) was 2 μL/mL in the study by Hasika Mith et al. [23]. However, the MIC of OGEO against *Listeria monocytogenes* (Gram positive) was higher than that of *S. typhimurium*. A possible reason for this finding could be the different inhibitory effects of OGEO on Gram positive and Gram negative. The hydrophobic mechanism of essential oils allowed them to enter the cell membrane and expand the permeability of the cells, thus enabling the release of the cell contents [24]. Gram positive have a thicker cell wall than Gram negative, making it more difficult for essential oil to enter and act [25]. We conjectured that OGEO in sub-MIC could also inhibit the growth of *S. putrefaciens* and reduce the biofilm formation.

### 2.3. Inhibition of Biofilm Formation Ability

Biofilms are a group of bacterial cells that are attached by secreted polymers, which can grow on both living and dead cell surfaces [26]. With this protective barrier, bacteria survived longer in harsh conditions. Therefore, this research further evaluated the effect of OGEO against *S. putrefaciens* biofilm formation.

**Figure 1:**
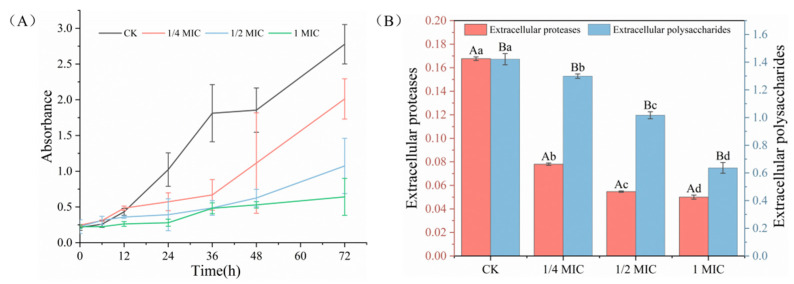
The biofilm formation (**A**) and extracellular polymeric substances (**B**) of *S. putrefaciens* treated with *Ocimum gratissimum* L. essential oil. Different letters indicate statistically significant differences (*p <* 0.05).

As shown in Figure 1A, incubation for 24 h resulted in significantly fewer biofilms in the OGEO-treated groups than in the CK group (*p* < 0.05). It indicated that biofilm formation was inhibited in a dose-dependent manner over the measured time. The results of this assay were in agreement with those of Martino et al. [27], who found that OGEO showed good biofilm inhibition against *Escherichia coli*, *Pectobacterium carotovorum* and *Pseudomonas aeruginosa* with a percentage inhibition of more than 58%.

The inhibition of biofilm production was most pronounced in the OGEO groups at 72 h incubation. Biofilm production in the 1/4× MIC, 1/2× MIC and 1× MIC groups was reduced by 27.54%, 61.30% and 76.90%, respectively, compared to the CK. Additionally, 1× MIC OGEO was the most effective in inhibiting biofilm formation. Snoussi et al. [28] also demonstrated that higher concentrations of OGEO provided better ability to resist biofilm formation. Additionally, 2× MIC of basil oil had an ability to inhibit around 30% of the biofilm of strains. The inhibition rate of biofilm reached 50% when the concentration was increased to the MBC. The suppression ratio of biofilm ranged from 55% to 87.45% when 50 mg/ml of *O. basilicum* essential oil was used to treat all strains. However, the formed biofilms were not completely removed, even at the higher concentration of 1× MIC.

Hydrophobicity is one of the most important determinants influencing bacteria adhesion and/or biofilm formation [29]. Previous studies had demonstrated that cell surface hydrophobicity of bacteria had a positive effect on bacteria adhesion and consequent biofilm formation. Changes in cell surface hydrophobicity had the potential to modify the formation of biofilms by altering the attachment of the strain to the polymer surface [30]. Thus, the addition of OGEO reduced the ability to form biofilms, which could be associated with the alteration of the hydrophobic properties of the *S. putrefaciens* isolate by OGEO.

### 2.4. Inhibition of Extracellular Polymeric Substances

Extracellular polymeric substances comprise polysaccharides, proteins, extracellular *DNA* (*eDNA*) and lipids. They are organic polymers of bacteria involved in bacterial cells’ interactions with the environment [31]. The rubber-like biofilms are secreted by the sessile cells and formed so that the structure has a high level of behavior and viscoelasticity [32]. It can be seen that the high degree of viscoelasticity causes the biofilm to adhere tightly to the vessel walls.

**Figure 2:**
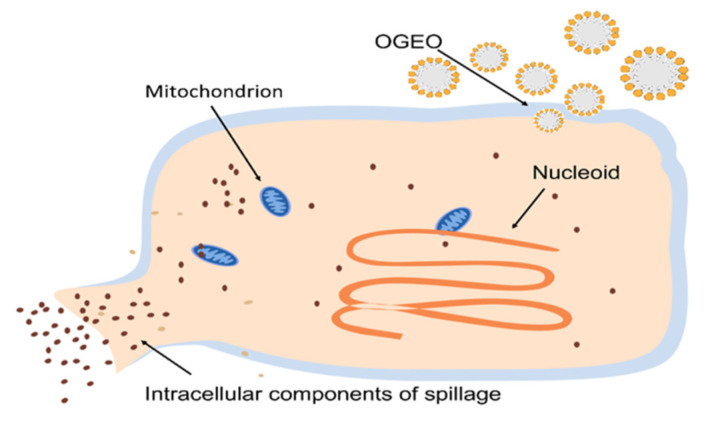
The antibacterial mechanism of *Ocimum gratissimum* L. essential oil against *S. putrefaciens* [34].

The inhibition trends of extracellular polysaccharide (EPS) and extracellular protease (EP) at different concentrations of OGEO are shown in Figure 1B. The EPS and EP contents of *S. putrefaciens* treated with OGEO were significantly reduced when compared with those of CK (*p <* 0.05). Furthermore, the inhibition rates of EPS and EP increased with increasing concentrations. As shown in Table 2, OGEO treatments at 1/4× MIC, 1/2× MIC and 1× MIC concentrations were negatively correlated with the inhibition rate of EPS (r = −0.9256 to −0.9966). However, the correlation of the relationship for biofilm EP (r = −0.9997 to −0.9998) was not significant (*p* > 0.05). The biofilms treated with 1× MIC OGEO resulted in a 55.26% and 70.18% decline in the EPS and EP contents of *S. putrefaciens*, demonstrating that OGEO inhibited the formation of EPS and EP. This was consistent with the finding of Wang et al. [33], who found that carvacrol effectively inhibited the secretion of EPS by *P. fluorescens*. As shown in Figure 2, the mechanism of action of OGEO against *S. putrefaciens* involved the disintegration of the cytoplasmic membrane, the release of the intracellular materials and an increase in the permeability of the cytoplasmic membrane. Under the action of OGEO against *S. putrefaciens*, the cell wall and membrane were destroyed. Then, a large number of electrolytes from *S. putrefaciens* permeated into the suspension, resulting in a change in the conductivity of the cells in the suspension. The intracellular ion balance played an important role in maintaining intra- and extracellular osmotic pressure. The loss of ions affected the intra- and extracellular osmotic pressure and caused the cells to swell and even rupture, so that the contents of the bacterium leaked out.

### 2.5. Inhibition of Motility

Motility (including swimming and swarming) causes the bacteria to migrate away from the initial area and the biofilm extension becomes larger [35]. In the experiment, the motility of *S. putrefaciens* was evaluated in the absence or presence of OGEO. The motility of *S. putrefaciens* was significantly (*p* < 0.05) affected by the OGEO in a concentration-dependent manner. Swimming motility is a flagellum-dependent movement of individual cells observed in semi-solid media [36]. As shown in Table 3, high concentrations of OGEO impaired the motility of *S. putrefaciens*. With the 1× MIC OGEO treatment, there was a 74.79% inhibition of swimming motility in *S. putrefaciens* compared with the CK group. Swarming was defined as the rapid, coordinated movement of bacteria on a semi-solid surface [37]. Swarming increased the surface colonization less than swimming. OGEO significantly reduced the swarming zone of the *S. putrefaciens* (*p* < 0.05). The presence of high levels of OGEO treatments greatly inhibited the mobility of bacteria, with a minimal diffusion diameter.

### 2.6. Hemolytic Activity Analysis

**Figure 3:**
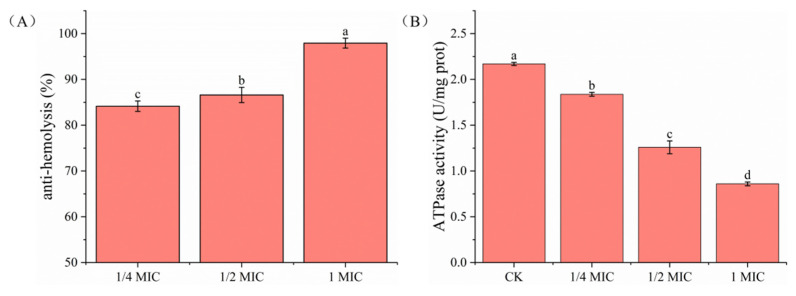
The hemolysis (**A**) and ATPase activity (**B**) of *S. putrefaciens* treated with *Ocimum gratissimum* L. essential oil. Different letters indicate statistically significant differences (*p <* 0.05).

Hemolytic activity is a characteristic of essential oils to bring about hemolysis. In other words, hemoglobin in the erythrocytes is released due to changes in membrane permeability [38]. The ability to cause hemolysis is common in the action of virulence factors in bacteria [39]. OGEO significantly reduced the hemolytic activity of *S. putrefaciens* at a concentration of 1× MIC (*p* < 0.05) (Figure 3A). The effect was more pronounced as the concentration increased. From 1/4 × MIC to 1× MIC, the antihemolytic activities increased from 84.15% to 97.93% for *S. putrefaciens*. The structure of the cell was disrupted and the permeability increased. The ability of OGEO was evaluated to inhibit hemolysis induced by hemolysin. The results showed that the different concentrations of OGEO significantly reduced the hemolytic activity of *S. putrefaciens* (*p* < 0.05). The production of beta-hemolysin by *S. putrefaciens* could lead to hemolysis [40]. OGEO at 1× MIC inhibited hemolysis by more than 95%. Thus, OGEO not only has antibacterial activity, but it also inhibits virulence factors when used at concentrations below the MIC. Soltani et al. [41] also demonstrated that rosemary essential oil (high 1,8-cineole content) could inhibit hemolysis. They found that rosemary essential oil repressed the activity of hemolysis of *Streptococcus iniae*.

### 2.7. ATPase Activity Analysis

ATP was necessary for the vital activity of the bacteria and the activity of mitochondrial ATPase could reflect the content of intracellular ATP, which could even cause cell death [42]. Figure 3B shows that the ATPase activity of *S. putrefaciens* decreased significantly (*p* < 0.05) after OGEO treatment and the ATPase activities decreased from 2.169 (CK) to 1.837 (1/4× MIC), 1.258 (1/2× MIC) and 0.859 (1× MIC). These results suggested that OGEO inhibited the ATPase of *S. putrefaciens*. Small hydrophobic molecules can cause non-specific inhibition of enzymes bound or buried in membranes, which was explained by the change in protein conformation. This mechanism might lead to inhibition of ATPase along with other enzymatic activities and alter the growth of bacteria [43]. It could be inferred that OGEO reduced the ATPase activity by impairing the function of the cell membrane barrier. In a previous study, it had been demonstrated that the active compounds of eugenol had an inhibitory effect on the ATPase of *E. coli* (Gram negative) [44]. In their experiment, they demonstrated that the inhibition of ATPase by eugenol was concentration-dependent. The finding explained the reason that high concentrations of OGEO possessed a better inhibition effect. Cui et al. [45] believed the reduction in ATPase levels was one of the main factors contributing to bacterial cell death. Mitochondria are the main source of ATP and inhibition of mitochondrial ATPase will lead to the inhibition of membrane ATPase and further lead to a decrease in the pH of the medium acidification. This phenomenon leads to a disruption of the permeability and structure of the cell membrane [42]. These results further confirmed that OGEO inhibited the ATPase activity of *S. putrefaciens* and impaired its metabolic capacity.

### 2.8. SEM and CLSM

**Figure 4:**
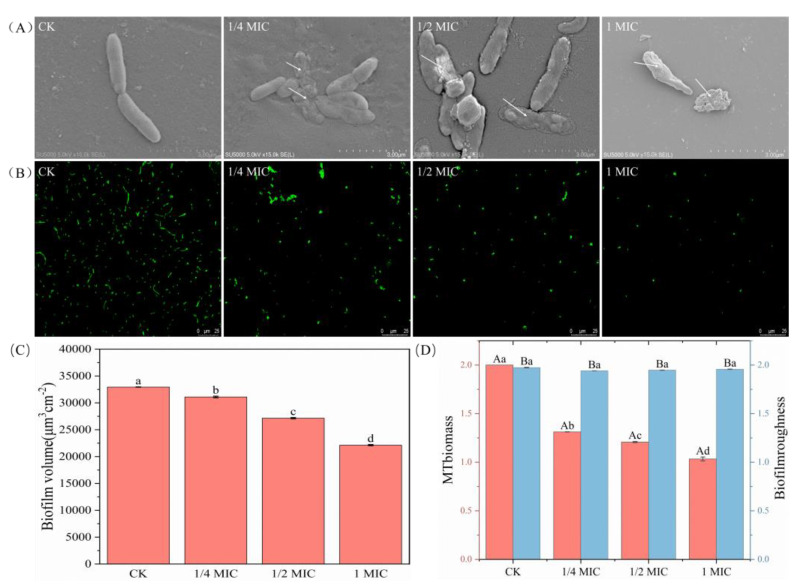
The SEM (**A**), CLSM (**B**), biofilm volume (**C**) and MTbiomass and biofilm roughness (**D**) of *S. putrefaciens* treated with *Ocimum gratissimum* L. essential oil. Different letters indicate statistically significant differences (*p <* 0.05).

Figure 4A shows that the OGEO-treated bacterial cells revealed grievous damage compared to that of CK. The cells of CK had intact cell morphology and formed a smooth and homogeneous biofilm. Nevertheless, the cells of the strains treated with OGEO were dry in shape, surface-depleted, disorganized and appeared to be aggregated. The structural integrity of the biofilm was clearly disrupted. The antibacterial components of OGEO could alter the outer membrane permeability and cell membrane function and cause leakage of intracellular substances [46]. OGEO caused *S. putrefaciens* to lose the intercellular connections in the biofilm. The structure of the biofilm was also disrupted in the case of treatment with OGEO, leading to roughness and shrinkage of the cells. The dead cells or cell fragments could be completely identified through pores in the cell membrane. The ability of OGEO to clear the biofilm was in large part due to eugenol, with disruption of cell-to-cell connections and cell lysis being the main forms of eugenol action [47]. The results of SEM in this experiment were consistent with the reported study. Changes in bacterial surface pathology and cellular damage were observed in *Escherichia coli* treated with eugenol [10].

CLSM results were presented in Figure 4B, where the number of *S. putrefaciens* was reduced after treatment with OGEO. It was found that the fluorescence of CK was in high quantity, and the distribution was close and uniform. However, the amount of fluorescence in the visible region decreased with increasing concentrations of OGEO, suggesting that OGEO had an inhibitory effect on biofilm formation in *S. putrefaciens*. ISA-2 analysis was used to convert the information from CLSM images into computable data, as shown in Figure 4C,D. Combining the ISA-2 analysis with the CLSM observations revealed some distinct features. Firstly, the biofilm volume of *S. putrefaciens* decreased by 5.62% (1/4× MIC), 17.60% (1/2× MIC) and 32.83% (1× MIC). Secondly, there was a significant reduction in biofilm biomass, especially between CK and 1/4 MIC. Thirdly, there was basically little change in biofilm roughness, but the treatment group had reduced roughness compared to CK. Wang et al. [4] showed that the biofilm roughness tested also increased significantly with increasing carvacrol concentration.

### 2.9. FTIR Analysis

**Figure 5:**
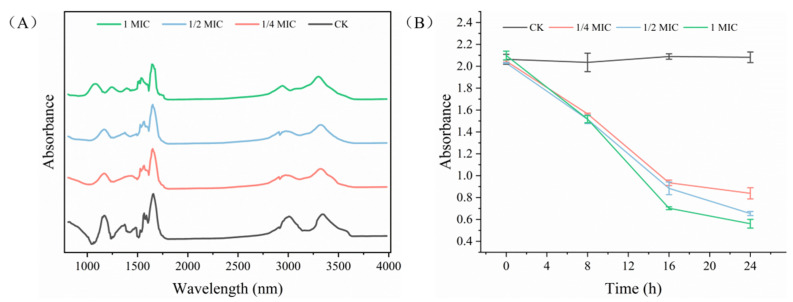
The Fourier transform infrared (FTIR) spectroscopy (**A**) and XTT (**B**) of *S. putrefaciens* treated with *Ocimum gratissimum* L. essential oil.

To further verify the inhibitory activity of OGEO against *S. putrefaciens*, the FTIR technique was used to detect the secondary structure of the biomacromolecular couples in *S. putrefaciens*. The distinct absorption bands identified around 3301 cm^−1^, 2938 cm^−1^, 1650 cm^−1^, 1535 cm^−1^, 1241 cm^−1^ and 1079 cm^−1^ corresponded to the deformation of the -OH stretching vibration, C-H stretching vibration, -CO stretching vibration, protein amide II, -SO stretching vibration and nucleic acid, respectively [48]. The following information can be obtained from Figure 5A: the bands of 1650 cm^−1^ and 1535 cm^−1^ were reduced, which meant that the protein content in the external environment of the bacteria increased. It was likely that the leakage occurred due to the disruption of the cell membrane. The cell membrane in the phospholipid structure of *S. putrefaciens* was disrupted by OGEO, as the data measured at 3301 cm^−1^ and 1241 cm^−1^ showed a decrease in the absorption peak. In addition to this, a clear reduction in the absorption peak at 1079 cm^−1^ occurred, indicating that nucleic acids in *S. putrefaciens* were leaking. All phenomena indicated that OGEO disrupted the cell membrane structure of *S. putrefaciens* and inhibited its growth. The most significant changes after treatment with OGEO occurred in the nucleic acids, followed by the cell membrane and protein. These results were also identical to the findings regarding cell membrane integrity.

### 2.10. XTT

The metabolic activity of cells in biofilms was assessed by using the XTT assay. At the 8th h, OGEO had a significant inhibitory effect on the cellular metabolic activity of *S. putrefaciens* (*p <* 0.05), but the difference in the inhibitory effect between different concentrations of OGEO was not significant (*p >* 0.05). After the 16th h, the inhibitory effect of OGEO with high concentrations on cellular activity became apparent and the best inhibition was shown at 1× MIC. OGEO effectively reduced the biomass of biofilms and impaired the metabolic activity of adherent cells formed in biofilms. The results were also supported by Almanaa et al. [49], who reported that essential oils were more potent inhibitors of biofilm-forming *Pseudomonas aeruginosa* (Gram negative). The result was a decrease in survival and an increase in dead cells, indicating that the EO was very effective in biofilm formation.

## 3. Materials and Methods

### 3.1. GC-MS Analysis

OGEO was bought at Chongqing Chunzhiyu Trading Company (Chongqing, China). OGEO was analyzed by GC-MS (Agilent 7890A-5975C, Santa Clara, CA, USA) equipped with capillary fused silica column HP-5MS (30m × 0.25 mm, 0.25 μm). The injector temperature was 250 °C, the detector temperature was 230 °C and the ion source temperature was 230 °C. The oven temperature was held at 45 °C for 1 min and then adjusted to 300 °C at 20 °C·min^−1^. The injection volume was 1 μL, the ionization energy was 70 EV and the scan range was 30–600 m^−1^z^−1^. The composition of the essential oils was determined by comparison with the NIST17 mass spectrometry library [50].

### 3.2. Bacterial Culture

*S. putrefaciens* (CICC 22940) was purchased from the China Center of Industrial Culture Collection (Beijing, China). The unactivated *S. putrefaciens* was kept at −80 °C. *S. putrefaciens* was activated in trypticase soy broth (TSB) before being used, treated at a temperature of 30 °C for 18 h. *S. putrefaciens* was inoculated into sterile TSB medium (HopeBio, Qingdao, China) at 1% (*v*/*v*) and then incubated at 30 °C until OD_600_ = 0.3. The incubated solute was used for the subsequent determination of the indicators.

### 3.3. MIC and MBC Measurements

OGEO was diluted from 256 μL/mL to 0.5 μL/mL by using TSB (two-fold dilution method). An amount of 256 µL of OGEO and 744 µL of 10% (*v*/*v*) Tween-80 were added to a sterile tube and mixed by shaking fully. An amount of 500 µL of the mixed solution was added to a sterile tube containing 500 μL of TSB, which was repeated until the concentration of OGEO was 0.5 µL/mL. Equal amounts (100 μL) of *S. putrefaciens* and diluted OGEO were added to a 96-well microtiter plate and mixed. Cultures were incubated at 30 °C for 24 h and then their growth was observed. The wells without observable bacterial colonies (concentration ≥ MIC) were incubated on TSA plates to determine the MBC of OGEO acting on *S. putrefaciens*. The inoculated TSA plates were incubated at 30 °C for 24 h. MIC is defined as the lowest OGEO concentration at which the suspension is clear and transparent (OD_600_ ≤ 0.05). For further assessment of bacterial growth, cultures without visible bacterial growth on the agar were determined as the lowest bactericidal concentration of OGEO and were defined as the MBC [51].

### 3.4. Quantification of Biofilms Using Crystal Violet Staining

Crystal violet staining was applied to quantify the biofilms [52]. Different concentrations of OGEO and the bacterial solution (1:1, *v*/*v*) were added to a 48-well microtiter plate and then incubated at 30 °C for 24 h. Biofilms were gently rinsed with PBS (0.01 M, pH 7.0, 4 °C) 3 times to remove the unattached cells. In the first case, biofilms were fixed for 30 min at 60 °C. The biofilm was treated with crystal violet (0.2%, *w*/*v*) to develop the color of the biofilm: the amount of dye used was 1 mL and the staining time was 15 min. At the end of the staining, the dye was used to remove the surface with PBS. Finally, 1 mL ethanol (95%, *v*/*v*) was added to dissolve the dye for 5 min. Absorbance was measured at 600 nm. The inhibition ratio was calculated as follows:

$$Inhibition\;ratio\;\left(\%\right)\;=\frac{OD_{600}\;of\;CK-OD_{600}\;of\;sample}{OD_{600}\;of\;CK}\times \;100$$

### 3.5. Extracellular Polysaccharide (EPS) Measurements

TSB broth with and without OGEO was inoculated in 1% bacterial culture and incubated (30 °C, 9 h). The supernatant (centrifugation at 706× *g* for 15 min) was mixed with 95% ethanol (1:3) and the precipitate was obtained by centrifugation at 706× *g* for 15 min. The precipitate was subsequently mixed with water, phenol solution (5%, *v*/*v*) and concentrated sulfuric acid in the ratio of 1:1:5 (*v*:*v*:*v*) and reacted for 20 min. The absorbance was detected at 490 nm [7].

### 3.6. Extracellular Protease (EP) Production Measurements

The EP assay was slightly modified according to previous studies [53]. The TSB broth with and without OGEO was inoculated in 1% *S. putrefaciens* and incubated at 30 °C for 9 h. At the end of bacterial cultivation, the suspension was centrifuged at 11,292× *g* for 1 min at 4 °C. Afterwards, the supernatant was mixed with PBS (containing 10 mg/mL Aspergillus powder) and incubated at 30 °C for 2 h. The supernatant was aspirated in a 96-well plate and then measured at 600 nm for absorbance to calculate the effect of protease inhibition.

### 3.7. Swimming and Swarming Motility Analysis

The motility analysis of *S. putrefaciens* was assessed on semisolid medium. An amount of 20 mL of medium (swimming with 0.3% agar and swarming with 0.5% agar) was poured into the sterile culture dishes. The final concentrations of OGEO in the dishes were made to be 0 (CK), 1/4× MIC, 1/2× MIC and 1× MIC, respectively. An amount of 5 μL of the bacterial suspensions (OD_600_ = 0.5) was dropped onto the plates individually and incubated at 30 °C for 20 h. The diameters were measured after incubation [54].

### 3.8. Hemolytic Activity Test

The method of hemolysis in this experiment was modified from a previous study [55]. Briefly, *S. putrefaciens* was incubated overnight (OD_600_ = 0.5) in TSB broth diluted 1:100 with or without OGEO. Sheep erythrocytes (MBcell, Seoul, Republic of Korea) were washed 3 times with sterile PBS and diluted in PBS (100 μL erythrocytes in 900 μL PBS). *S. putrefaciens* cultured for 18 h was centrifuged (3416× *g*, 10 min) and 250 µL of the supernatant was added to 1 mL of diluted erythrocytes. The final mixture was incubated at 30 °C for 30 min to measure the hemolytic activity. The supernatant was obtained by centrifugation at 3416× *g* for 10 min. Finally, the absorbance of the supernatant was measured at 450 nm.

### 3.9. Determination of Extracellular ATPase Concentration

*S. putrefaciens* suspension was prepared. Then, 1/4× MIC, 1/2× MIC and 1× MIC concentrations of OGEO were added to each of the tested strains. After incubation, all samples were incubated at 30 °C for 30 min and centrifuged at 3416× *g* for 5 min. The collected supernatant was stored on ice plates. the concentration of extracellular ATPase was determined using the ATPase Assay Kit (Beyotime Bioengineering Institute, Shanghai, China) [5].

### 3.10. Scanning Electron Microscopy (SEM) Analysis

First, sterile slides were placed in 48-well plates. Then, the biofilm was formed by inoculating the cultured *S. putrefaciens* in wells containing different concentrations of OGEO for 22 h at 30 °C. Then, they were washed 3 times (with PBS) and fixed with a freshly prepared 2.5% glutaraldehyde. The fixed biofilm was washed with PBS and it was dehydrated using ethanol (30%, 50%, 70%, 90%) for 15 min. The dried biofilm was gold-sprayed and then observed by SEM (S-3400, Hitachi, Tokyo, Japan) [56].

### 3.11. Confocal Laser Scanning Microscopy (CLSM) Analysis

*S. putrefaciens* was incubated in a 48-well plate containing glass slides for 22 h to establish biofilms. To remove floating material, we used the method of washing the plates 3 times with PBS. The formed biofilms were set rigidly in place in 4% glutaraldehyde for 30 min and then washed (with PBS). Subsequent staining was performed with 1× SYBR Green I (Sangon Biotech, Co., Ltd., Shanghai, China) staining solution in the dark for 30 min. The biofilm formation was observed with CLSM (LSM710, Carl Zeiss AG, Jena, Germany) using a 63×/1.40 oil objective at 488 nm excitation. Parameters, such as MTbiomass, were evaluated using ISA-2 software [57].

### 3.12. XTT Content Measurements

The XTT content was slightly modified according to the method of Guo et al. [57]. The reduction in XTT is indicated by the reduction in metabolically active cells measured with the Cell Proliferation Kit II (XTT) (Sigma-Aldrich, St. Louis, MO, USA). The XTT labeling reagent and the electron coupling agent need to be melted in a water bath at a temperature of 37 °C. The XTT reagent is mixed with the XTT labeling reagent and the electron coupling agent. The biofilm of the test strain was stained with crystal violet. Then, 100 μL PBS and 50 μL XTT reagent were added to each well and incubated in a dark room at 30 °C for 24 h. After incubation, the absorbance was measured at 450 nm.

### 3.13. Fourier Transform Infrared (FTIR) Spectroscopy

The suspensions with final concentrations of 0× MIC (CK), 1/4× MIC, 1/2× MIC and 1× MIC content of OGEO were subjected to subsequent treatment. The precipitate was collected by incubation at 30 °C for 12 h and centrifuged at 1016× *g* for 10 min. The precipitate obtained was washed 3 times with PBS, freeze-dried and then tested by FTIR spectroscopy (Nicolet, Thermo Fisher Scientific, Waltham, MA, USA). To obtain good spectra, 32 scans were performed at a resolution of 4 cm^−1^ over a frequency range of 800–4000 cm^−1^ [58].

### 3.14. Statistical Analysis

All experiments were performed in three replicates. Results were shown as mean ± standard deviation (*n* ≥ 3). Data were subjected to statistical ANOVA using SPSS 22.0. Graphs were plotted using Origin 2018. *p <* 0.05 was considered significant.

## 4. Conclusions

The MIC and MBC of OGEO against *S. putrefaciens* were both 0.1%. Results obtained from SEM had shown that OGEO disrupted the integrity of the cell membrane and wall, leading to leakage of nucleic acids and proteins. The results of FTIR further confirmed the antibacterial properties of OGEO, which disrupted the phospholipid structure of the membrane and inhibited the growth of the strain. Moreover, high concentrations of OGEO reduced the number of biofilms produced by more strains and inhibited biofilm movement to a greater extent, effectively demonstrating the attenuating effect of OGEO on biofilm formation. These results suggested that OGEO could be a promising natural preservative for food products, with great research value in inhibiting microbial growth and extending shelf life.

## Figures and Tables

**Table 1:** The main active ingredient of *Ocimum gratissimum* L. essential oil. each compound is averaged after three determinations and the concentration is expressed as a percentage of the total area normalized by information systems technology and design (ISTD).

NO.	Main Compounds	Retention Time (min)	Retention Index	Peak Area (%)	CAS
1	Heptane, 2,2,4,6,6-pentamethyl-	7.478	1197	0.23	13475-82-6
2	Cyclotetrasiloxane, octamethyl-	7.825	1227	0.17	556-67-2
3	Heptane, 4-ethyl-2,2,6,6-tetramethyl-	7.887	1233	0.03	62108-31-0
4	2,2,4,4, -Tetramethyloctane	8.112	1252	0.07	62183-79-3
5	β-pinene	8.25	1263	0.45	17301-28-9
6	Linalool	9.358	1360	1.09	78-70-6
7	Cyclopentasiloxane, decamethyl-	10.08	1426	0.74	541-02-6
8	Estragole	10.962	1509	4.87	140-67-0
10	α-Cubebene	13.000	1718	0.06	17699-14-8
11	Eugenol	13.161	1735	51.88	97-53-0
12	3-Allyl-6-methoxyphenol	13.381	1758	3.15	501-19-9
13	Caryophyllene	13.976	1817	9.57	87-44-5
14	trans-α-Bergamotene	14.109	1829	0.56	13474-59-4
15	Humulen	14.34	1851	0.93	24405-93-4
16	1,4,7,-Cycloundecatriene, 1,5,9,9-tetramethyl-, (1Z,4Z,7Z)-	14.406	1857	2.34	400822-79-9
17	(+)-δ-Cadinene	14.662	1881	0.74	483-76-1
18	Cycloheptasiloxane, tetradecamethyl-	14.792	1893	1.32	107-50-6
19	δ-cadinene	15.238	1931	0.25	483-76-1
20	4-allyl-2-methoxyphenyl acetate	15.445	1950	0.13	93-28-7
21	Caryophyllene oxide	16.027	1999	1.05	1139-30-6
22	Humulene	16.203	2014	3.23	6753-98-6
23	Cyclooctasiloxane, hexadecamethyl-	16.785	2061	0.08	556-68-3
24	Androstan-17-one, 3-ethyl-3-hydroxy-, (5 α)-	16.887	2070	2.37	57344-99-7
25	Patchouli alcohol	16.947	2074	3.35	5986-55-0
26	Undecane, 3,6-dimethyl-	17.3118	2298	0.09	18172-67-3
27	β-myrcene	19.5734	2473	0.02	123-35-3
28	Bikaverin	25.054	2665	0.76	33390-21-5
29	3-Hexen-1-ol, (Z)-	27.6961	2795	0.49	928-96-1
30	Acetic acid	29.3855	2933	2.89	64-19-7
31	5-Methyl-2-furfural	32.8607	3197	0.01	620-02-0
32	Phthalic acid, mono(o-methylbenzyl) ester	34.5087	3376	0.50	4619-49-2
33	α-Amorphene	36.0394	3487	0.01	23515-88-0
34	γ-Cadinenema	38.3287	3658	0.73	39029-41-9
35	Safrole	39.6732	3720	0.46	94-59-7
36	Phenol, 2-methoxy-4-(2-propenyl)-	43.4243	4089	0.58	97-53-0
37	Patchouli alcohol	44.3276	4173	0.74	5986-55-0
38	1-Cyclopentenecarboxylic acid, 2-methyl-3-vinyl-, 4′-fluorophenyl ester	45.1205	4298	0.11	1000158-81-1
39	Chavicol	45.6515	4367	0.01	501-92-8
40	Isoaromadendrene epoxide	46.5961	4449	0.06	1000159-36-6
41	6-Methoxy-3-methylbenzofuran	48.0786	4667	0.03	29040-52-6
42	Triacontane	48.1958	4713	3.85	638-68-6

**Table 2:** The correlation between the concentration of *Ocimum gratissimum* L. essential oil and the inhibitory effect of extracellular polymers.

Index	CK	1/4× MIC	1/2× MIC	1× MIC
EPS	-	−0.9256	−0.9909	−0.9966
EP	-	−0.9997	−0.9998	−0.9997

**Table 3:** Swimming and swarming motility of *S. putrefaciens* in the presence of *Ocimum gratissimum* L. essential oil at different concentrations.

Samples	Swimming (mm)	Swarming (mm)
CK	80.58 ± 0.47	58.42 ± 0.64
1/4 MIC	52.14 ± 0.34	35.25 ± 0.20
1/2 MIC	16.37 ± 0.25	22.98 ± 0.92
1 MIC	9.34 0.11	14.73 ± 0.57

## Data Availability

All data generated or analyzed during this study are included in this published article.
